# Stacking fault aggregation during cooling composing FCC–HCP martensitic transformation revealed by *in-situ* electron channeling contrast imaging in an Fe-high Mn alloy

**DOI:** 10.1080/14686996.2021.1877570

**Published:** 2021-03-15

**Authors:** Motomichi Koyama, Misaki Seo, Keiichiro Nakafuji, Kaneaki Tsuzaki

**Affiliations:** a Institute for Materials Research, Tohoku University, Sendai, Miyagi, Japan; b Elements Strategy Initiative for Structural Materials (ESISM), Kyoto University, Kyoto, Japan; c Faculty of Engineering, Kyushu University, Fukuoka, Japan; d Research Center for Structural Materials, National Institute for Materials Science, Tsukuba, Japan

**Keywords:** Stacking fault, martensitic transformation, austenitic steel, electron channeling contrast imaging, *in situ* electron microscopy, Metallic materials, TEM, STEM, SEM

## Abstract

To understand the mechanism of FCC–HCP martensitic transformation, we applied electron channeling contrast imaging under cooling to −51°C and subsequent heating to 150°C. The stacking faults were randomly extended and aggregated during cooling. The stacking fault aggregates were indexed as HCP. Furthermore, the shrink of stacking faults due to reverse motion of Shockley partials was observed during heating, but some SFs remained even after heating to the finishing temperature for reverse transformation (A_f_: 104°C). This fact implies that the chemical driving force of the FCC/HCP phases does not contribute to the motion of a single SF but works for group motion of stacking faults.

## Graphical abstract

**Figure:**
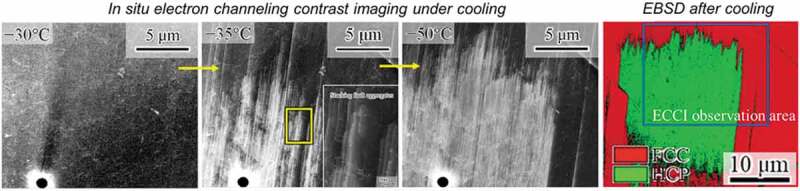

## 1. Introduction

Electron channeling contrast imaging (ECCI) enables observations of dislocations and stacking faults [1–3]. Therefore, the ECCI technique has been used for the characterization of dislocation-driven phenomena such as the plastic deformation of metals [4,5]. Moreover, the ECCI technique has been recognized as a new pathway for *in situ* observations [6–8] because of two major points. First, bulk specimens are available for ECCI. Although ECCI is a surface observation technique, the deformation constraint of the bulk specimen is significant, particularly when in-plane deformation phenomena are the target. Second, observations at multiple scales from tens of nanometers to sub-millimeters can be achieved easily, which enables us to observe the surrounding microstructure over a grain-size scale and target microstructure on a dislocation-resolved scale simultaneously. Hence, ‘lattice-defect-resolved’ and ‘multi-scale’ observations in ‘bulk’ specimens are crucial for *in situ* deformation experiments in metals.

From the viewpoint of dislocation-driven phenomena, martensitic transformation is another important topic, which requires *in situ* experiments to uncover the mechanisms. For instance, the martensitic transformation from a face-centered cubic (FCC) to a hexagonal close-packed (HCP) structure plays multiple roles in the functional [9–11] and mechanical properties [12–15] of Co and Fe alloys. Therefore, the mechanisms of forward and reverse transformation of HCP martensite have been investigated widely through *in situ* transmission electron microscopy (TEM) [16–19]. As a representative growth mode of HCP martensite, random aggregation of intrinsic stacking faults has been reported. Specifically, bundles of intrinsic stacking faults with a thickness of 1–2 nm form and then multiple bundles aggregate randomly to form thicker bundles [20]. In addition to the previous TEM works, some additional *in situ* experiments under deformation constraint with information pertaining to the surrounding microstructure would be helpful to understand additional details of the martensite formation process, specifically, *in situ* ECCI can support the proposed mechanisms. In this study, we present the results of *in situ* ECCI during cooling and subsequent heating to characterize the thermally induced extension and shrink of the intrinsic stacking faults that compose FCC–HCP martensitic transformation.

## 2. Materials and methods

In this study, an ingot of Fe-15Mn-10Cr-8Ni alloy, which shows HCP martensitic transformation [14,21], was prepared by vacuum induction melting. The ingot was hot-forged and rolled at 1000°C. Then, the rolled bar was homogenized at 1200°C for 1 h, followed by water-quenching. A specimen with gauge dimensions of 1.2 mm thickness, 4.0 mm width, and 10 mm length was cut from the bar using electric discharge machining. To limit the transformation path of the martensite, the specimen was pre-deformed by 4% strain and subsequently heated to 150°C for 10 min in vacuum. The starting and finishing temperatures for reverse and forward transformations, A_s_, A_f_, and M_s_, of the 4%-strained specimen were confirmed to be 70°C, 104°C, and −33°C, respectively, by means of differential scanning calorimetry at heating and subsequent cooling rates of 20°C/min. Because of the presence of nucleation sites and internal stresses, the transformation path of the pre-strained/heated specimen was expected to be limited during the cooling and heating for *in situ* ECCI. In addition, we selected a grain for which no intensive surface relieves formed after the 4% strain. Because a previous study indicated that all grains showed martensite after 4% strain [22], the absence of surface relieves indicates that martensite in the grain formed by in-plane shear of the specimen. Through the selection of this grain, this experiment attained the condition of a specimen interior with a minimal effect of surface relieve on electron channeling contrast.

After heating for reverse transformation, the specimen was mechanically polished with colloidal silica with a particle size of 60 nm. The polished specimen was set to a cooling stage (produced by the Mel-Build Corporation, Japan [23]) to conduct *in situ* ECCI during cooling from room temperature (20°C) to −51°C in the scanning electron microscope (Carl Zeiss Co., Ltd., Germany). The temperature was held within an accuracy of ±0.1°C for each observation. After the cooling experiment, the specimen surface was re-polished slightly with colloidal silica to remove contaminants such as hydrocarbons. Then, an electron backscatter diffraction (EBSD) measurement was carried out to identify the crystallographic orientation and phase. To investigate the reverse transformation as well, the same specimen was next set to a heating stage (produced by TSL Solutions, Japan [24]) for *in situ* ECCI. The specimen was heated from 20°C to 150°C in a microscope chamber. The acceleration voltage, probe current, and working distance for ECCI were set to 30 kV, 10 nA, and 3–4 mm, respectively. The EBSD measurement was carried out at 20 kV and 10 nA with a working distance of 15 mm. The beam step size was set to 50 nm. In the present observation, we first selected a grain where dark contrast appeared. The dark contrast in ECC images implies that the channeling condition is nearly satisfied. When the stacking fault and thin HCP martensite plate were characterized, the deviation parameter *w* [1,2] in the local region was confirmed to be positive by the EBSD measurement. Therefore, the present ECCI was not controlled [1] or accurate [25] ECCI in which the specimen inclination angle was determined based on the surface crystallographic orientation obtained prior to the operation of ECCI.

## 3. Results and discussion

**Figure 1.**
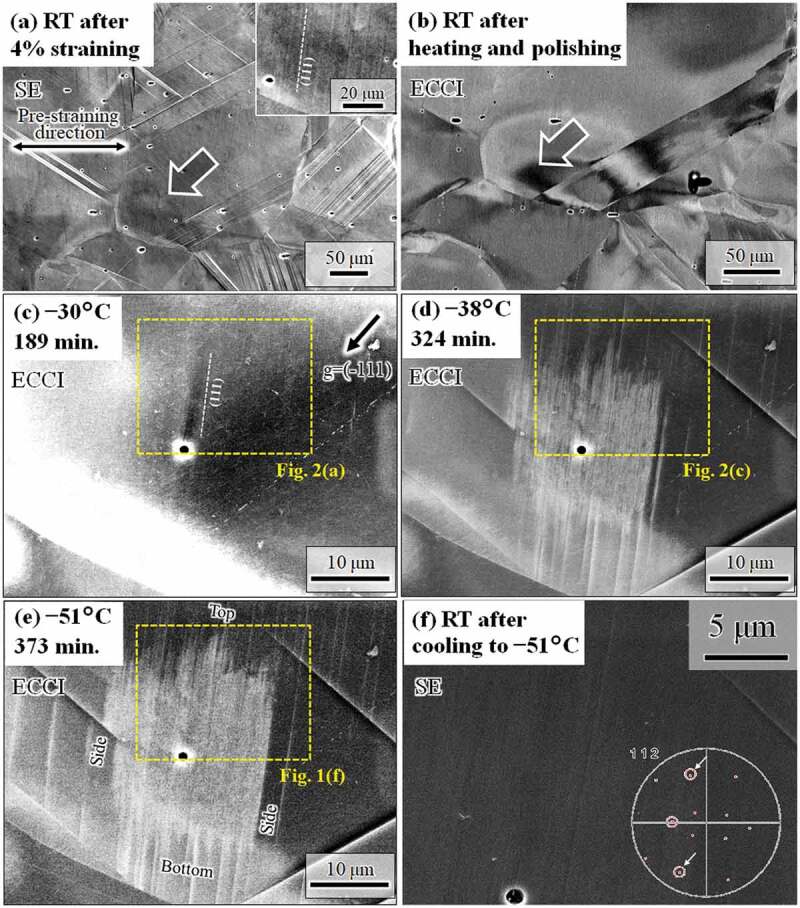
Set of images taken at an identical region. (a) Surface relieves after 4% straining. (b) Presence of internal stress before cooling. ECC images taken during cooling at (c) −30°C, (d) −38°C, and (e) −51°C. (f) SE image corresponding to the region highlighted in (e). The times indicate the total experimental time from the initiation of cooling. The black dots in the images are pores that would arise from the removal of inclusions during mechanical polishing

As shown in Figure 1(a), only slight surface relieves were observed in the target area pointed by the arrow. The inset in the figure indicates the presence of shallow surface relieves along a single (111) plane, indicated by the dotted white line, i.e., near-in-plane dislocation-driven shear. After heating and polishing, the ECC image, shown in Figure 1(b), revealed a contrast gradient near the grain boundary of the target area, which indicates the presence of internal stress [8,26]. In addition, the target area shows a black contrast, which indicates low backscatter electron intensity. That is, the channeling condition was nearly satisfied in the grain interior [1,3,27]. In Figure 3(c), locally different contrast along the (111) plane was observed, which was perhaps due to the residual stress resulting from the pre-strain-induced dislocation motion on the (111) plane and subsequent unloading. In the target grain, a contrast change appeared along the (111) and (−111) planes after cooling from −30°C (Figure 1(c)) to −38°C (Figure 1(d)). The contrast change is attributed to motion of Shockley partials on the respective planes. The white contrast on the (111) plane, which is the primary microstructural feature, grew from the grain interior to the grain boundary, and it became clear with further cooling (Figure 1(e)). The side edges of the white contrast were along the (111) plane, and the top and bottom edges showed zigzag features. Even after the formation of numerous plane defects, the surface relieves were very shallow (Figure 1(f)), similarly to the situation after pre-straining (Figure 1(a)). The shallow surface relieves can be created by two possibilities: (1) motion of Shockley partials with a Burgers vector aligned perpendicular to the normal of the specimen surface and (2) combination of the motion of Shockley partials with different Burgers vectors on the (111) plane. The inset of Figure 1(f) shows the <112> orientations in stereographic projection, which was obtained by EBSD at room temperature. For instance, the two <112> orientations on the (111) plane indicated by arrows are the FCC–HCP transformation path that could create the shallow surface relieves. Because the surface relieves are shallow, the ECC is attributed to internal elastic strain around the plane defects rather than the surface effects arising from surface relieves. An important finding here is that the microstructural change occurred preferentially at the specific site of the grain interior where pre-existing dislocations and internal stress were present. Furthermore, the side edges of the white contrast were parallel to the surface relieves that had been induced by the pre-strain shown in Figure 1(a). These facts indicate that the pre-existing dislocations and internal stress acted as the nucleation sites and driving force, respectively.

**Figure 2.**
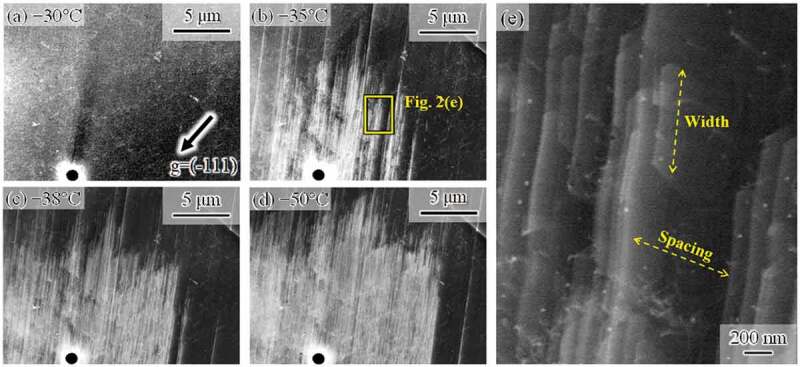
High-magnification ECC images taken during cooling at (a) −30°C, (b) −35°C, (c) −38°C, and (d) −50°C. (e) Magnified image of (b)

At the identical location during the same cooling process, high-magnification images were also acquired, as shown in Figure 2. The plane defects formed by cooling from −30°C (Figure 2(a)) to −38°C (Figure 2(b)) were composed of very thin plates along the (111) plane. The positions of the plate tips were random, which resulted in the zigzag feature. The density of plates increased with cooling (Figure 2(c)), and some plates aggregated with each other (Figure 2(d)). The higher magnification image (Figure 2(e)) indicates that the spacing and width of the plates were also random. According to previous studies, a very thin plate along {111} is a thin HCP-martensite plate [20] or a stacking fault [28]. These observation results are fully consistent with those of a previous study on deformation-induced FCC–HCP martensitic transformation of an Fe-Mn-Si-based shape memory alloy [20]. In addition, the randomness of the spacing and width of the very thin plates can be explained as follows. For instance, the HCP phase can nucleate via dissociation and reaction of dislocations (e.g [28].). Because the present specimen was strained and heated before cooling, numerous dislocations and stacking faults pre-existed along the specific slip plane where the surface relieves shown in the inset of Figure 1(a) formed. Therefore, the nucleation of the HCP plates could densely occur, and their thickening resulted from aggregation of the plates, as observed in Figure 2. The growth behavior of the HCP plates is dependent on the degree of the local repulsive stress arising from the pre-existing lattice defects, which would be random as long as the distribution of pre-existing defects is random.

**Figure 3.**
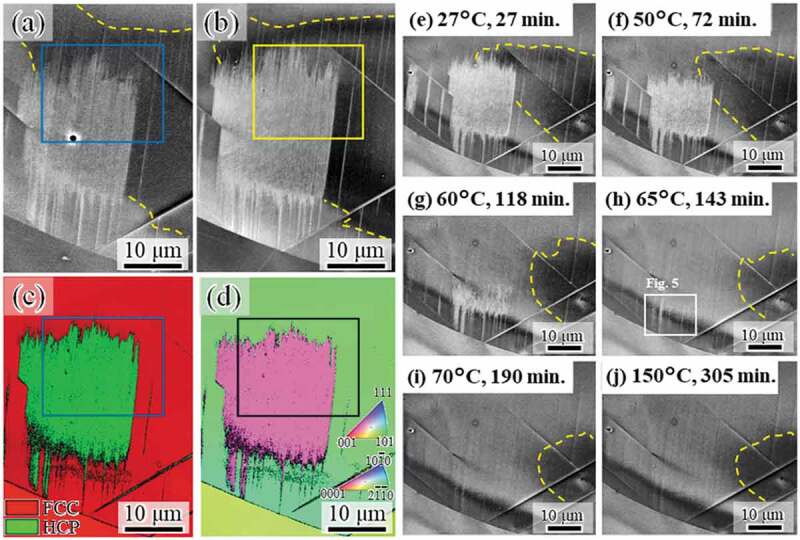
ECC images (a) before and (b) after slight mechanical polishing taken at RT after cooling to −51°C. (c) Phase and (d) ND-IPF maps of the same area as in (b). ECC images taken during heating at (e) 27°C, (f) 50°C, (g) 60°C, (h) 65°C, (i) 70°C, and (j) 150°C. The times indicate the total experimental time from the initiation of heating. The region highlighted by dashed lines shows a significant contrast gradient that indicates internal stress. The region highlighted by squares in (a–d) indicates the identical area used for the high-magnification observation shown in Fig. 4

To characterize reverse transformation, the identical microstructure was further analyzed after heating to room temperature and during further heating to 150°C. First, we confirmed that no significant change in the cooling-induced microstructure occurred during heating from −51°C to room temperature by comparing Figures 1(e) and Figures 3(a). Next, to remove contamination, which formed during the *in situ* observation during cooling, the specimen surface was mechanically polished slightly. The size and morphology of the microstructure showed almost no change after mechanical polishing, although the tip positions of the martensite plates and stacking faults changed slightly, as shown in Figure 3(a,b). That is, the mechanical polish did not alter the type of microstructure and crystallographic orientation. Through an EBSD measurement, the white contrast formed during cooling was identified as the HCP phase with a single crystallographic orientation (Figure 3(c,d)). The HCP martensite reversely transformed to FCC, as shown in Figure 3(e–j). In addition, the contrast gradient, which indicates internal stress, was observed, as highlighted by the yellow dashed lines in Figure 3(a–j). The internal stress was not observed at −51°C, as shown in Figure 1(e), which indicates that the internal stress around the martensite formed during heating to room temperature, perhaps due to the difference in thermal expansion rate between the FCC matrix and HCP martensite. The area where the internal stress existed decreased with the progress of the reverse transformation, and it did not change with further increase in temperature after completion of the reverse transformation (Figure 3(i,j)), i.e., the reverse transformation accommodated the internal stress. Note that the reverse transformation in the region with internal stress almost completed at 70°C, which corresponds to the A_s_ at which the reverse transformation starts macroscopically. This indicates that the locally existing internal stress effectively acted as a driving force for the reverse transformation, which corresponds to a reduction in internal stress. This observation is evidence that the local back stress acting on Shockley partials is significant for controlling the reverse transformation kinetics, which is important for improving the shape memory effects associated with the FCC–HCP martensitic transformation [29].

**Figure 4.**
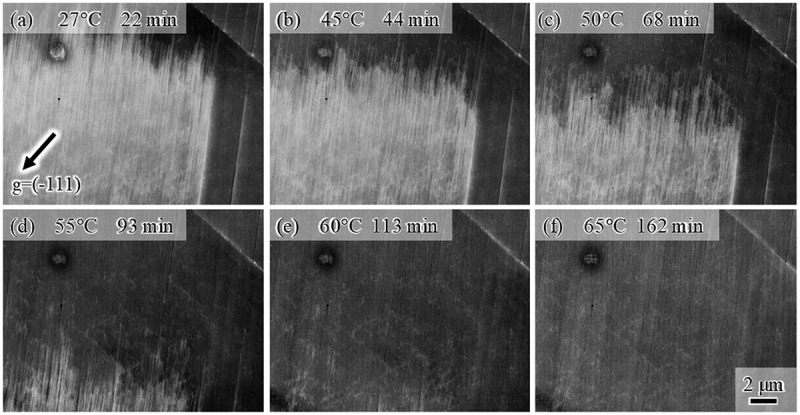
High-magnification ECC images taken during heating at (a) 27°C, (b) 45°C, (c) 50°C, (d) 55°C, (e) 60°C, and (f) 65°C

**Figure 5.**
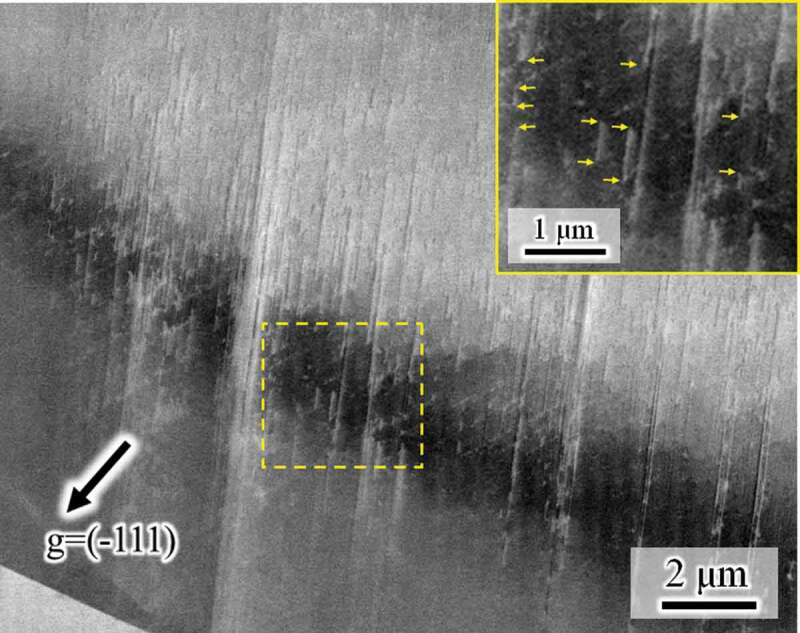
Another high-magnification image of the location highlighted in Fig. 3(h). The inset indicates a more highly magnified image showing stacking faults. The yellow arrows indicate tips of some plates, which demonstrates that the apparently plate-like microstructures are composed of discontinuous stacking faults

Figure 4 shows high-magnification images taken during heating. The plate density in the martensite decreased with temperature (Figure 4(a–c)). Then, the size of the martensite decreased (Figure 4(c–f). Similarly to the case of the forward transformation, the tips of the plates composing the HCP martensite are not ordered, i.e., the top and bottom edges of the martensite showed zigzag features, irrespective of temperature. This fact indicates that the reverse motion of Shockley partials for each plate is controlled by the local back stress acting on each dislocation, rather than by the group motion as single martensite. Furthermore, line marks remain even after heating to 150°C, which is much higher than the A_f_ temperature (Figure 3(j)). As illustrated in Figure 4(e,f), a considerable number of line marks were observed, and they were very fine. To characterize the line marks, we observed a slightly different region with a better diffraction condition at 65°C (Figure 5). The line marks consisted of discontinuous plate-like microstructures along the (111) plane. Because HCP martensite shows continuous plate-like features when forming in a single variant, the discontinuous plate-like microstructure is a stacking fault. Accordingly, the fine line marks remaining after the reverse transformation arose from residual stacking faults that formed during cooling. The fact that the line marks consisting of stacking faults remained even after heating to A_f_ (Figure 3(j)) indicates the following interesting point. Stacking faults, which did not compose the bundle, could not obtain a significant driving force for its full shrinkage by heating to A_f_. That is, a single stacking fault is not regarded as the HCP phase, and the chemical driving force of FCC/HCP acts for the group motion of Shockley partials.

## 4. Conclusions

In this study, we successfully applied *in situ* multi-scale ECCI under cooling and heating to characterize the forward and reverse FCC↔HCP martensitic transformations. The present method enabled stacking-fault-resolved characterization of the martensitic transformation sequence after the nucleation process in the bulk specimen. Specifically, random aggregation and shrink of stacking faults were observed in an Fe-Mn-based alloy containing numerous lattice defects. According to the EBSD analysis, the bundle of stacking faults was indexed as the HCP phase. The extension and aggregation behaviors of each stacking fault were random, and the shrink of the stacking faults was similar to the extension behavior. A significant factor affecting the random motion of stacking faults was the microstructural internal stress. Furthermore, some stacking faults that formed during the forward martensitic transformation remained even at temperatures above A_f_, which indicates that the chemical driving force of FCC/HCP does not contribute to the motion of single stacking faults and can work for group motion of stacking faults.
